# Acute appendicitis presenting as small bowel obstruction: two case reports

**DOI:** 10.1186/1757-1626-2-9106

**Published:** 2009-11-28

**Authors:** Sanjay Harrison, Kamal Mahawar, Dougal Brown, Leslie Boobis, Peter Small

**Affiliations:** 1 Department of General Surgery, Sunderland Royal Hospital, Kayll Road, Sunderland SR4 7TP, UK; 2 Department of Radiology, Sunderland Royal Hospital, Kayll Road, Sunderland SR4 7TP, UK

## Abstract

Acute appendicitis is a common surgical problem however the diagnosis is often overlooked when it presents as a small bowel obstruction. In this report we present two cases of elderly patients who presented with small bowel obstruction and raised inflammatory markers. Both patients were successfully treated with a laparotomy, adhesiolysis and appendicectomy and went on to make a good recovery.

## Introduction

Acute appendicitis may rarely present as small bowel obstruction [1]. The small bowel obstruction in such cases may be mechanical or due to ileus [2]. The clinical features of small bowel obstruction may dominate the clinical picture and mask appendicitis. This can pose a considerable diagnostic dilemma, especially in the elderly patients. We present here two cases of acute appendicitis presenting clinically as mechanical small bowel obstruction.

## Case presentations

### Case One

**Figure 1:**
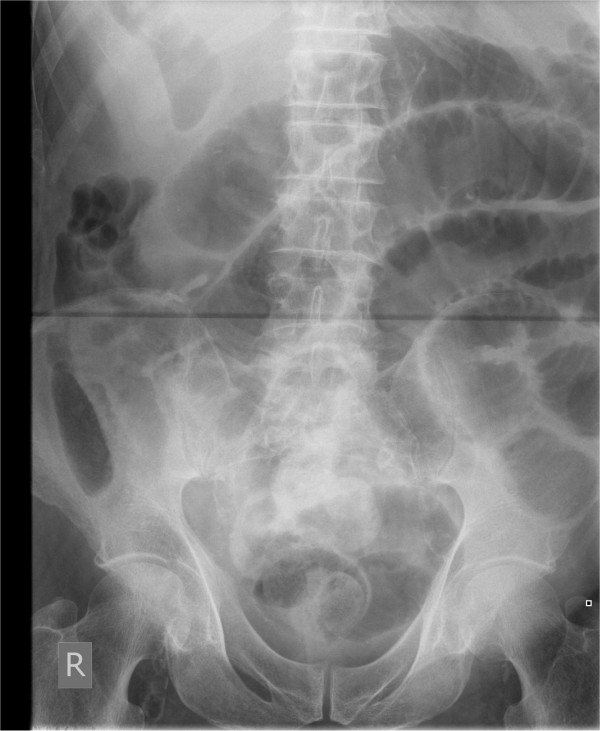
**Abdominal X-ray of patient 1 showing dilated loops of small bowel**.

**Figure 2:**
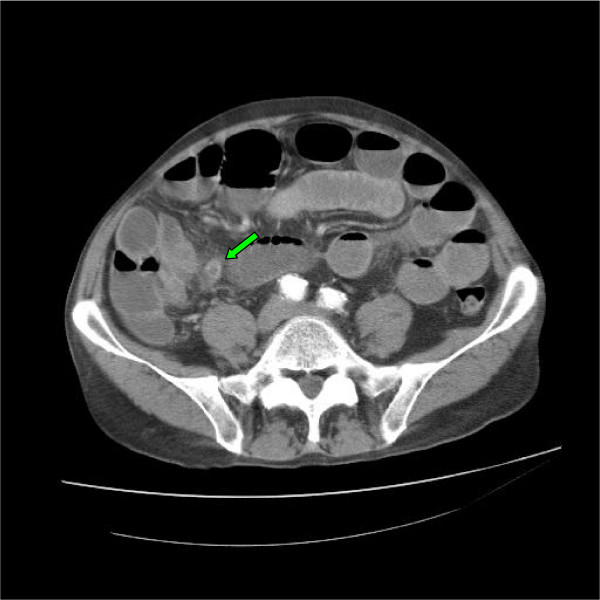
**Abdominal CT image of patient 1**. This image shows the presence of an inflammatory process in the right iliac fossa (green arrow).

A 62 year old lady presented with a four day history of abdominal pain, nausea, and vomiting. There was no history of prior abdominal surgery. On examination, she was mildly tachycardic with abdominal distension and poorly localized mild abdominal tenderness. Her routine blood investigations revealed a raised white cell count and C-reactive protein of 15.42 × 10^9^/L and 362 mg/L respectively. Her renal function was deranged with a creatinine of 251 μmol/L and a urea of 14.8 mmol/L. An abdominal film (Figure 1) showed multiple dilated loops of small bowel prompting a computerised tomography (CT) scan of her abdomen which revealed the presence of an inflammatory process in the right iliac fossa with an oedematous appendix and partial obstruction of the proximal small bowel loops (Figure 2). As the CT scan findings were highly suggestive of acute appendicitis, she was resuscitated overnight and an appendicectomy and adhesiolysis was performed the next day via a midline laparotomy. The diagnosis of appendicitis was confirmed at laparotomy and subsequently on histological examination of the removed appendix. She made a slow but complete recovery.

### Case Two

**Figure 3:**
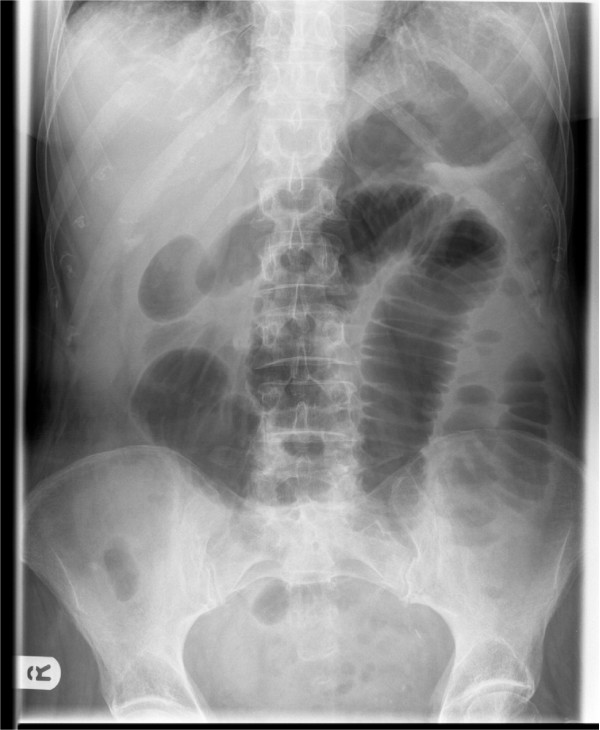
**Abdominal X-ray of patient 2 showing evidence of small bowel obstruction**.

**Figure 4:**
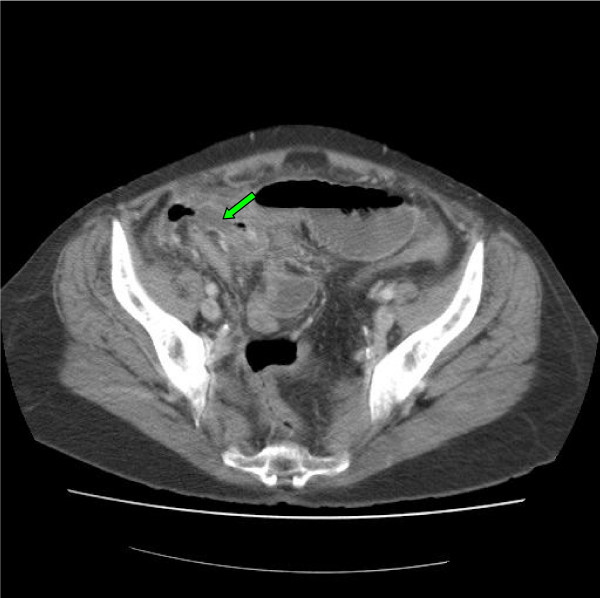
**CT of the abdomen of patient 2 showing the inflamed appendix (green arrow)**.

An 83 year old man was admitted with a four day history of worsening right sided abdominal pain associated with vomiting and loose stools. On examination, he was found to have mild abdominal distension and minimal central abdominal tenderness. Bowel sounds were exaggerated. Routine blood investigations revealed a raised white cell count and C-reactive protein levels of 12.83 × 10^9^/L and 275 mg/L respectively. His renal function was abnormal with a creatinine of 137 μmol/L and a urea of 15.8 mmol/L. An abdominal film (Figure 3) revealed prominent small bowel loops. A subsequent CT scan of his abdomen revealed dilated small bowel loops with associated inflammatory changes in the right iliac fossa (Figure 4). The findings were reported to be consistent with partial small bowel obstruction due to acute appendicitis. He underwent a laparotomy, adhesiolysis and appendicectomy. Postoperative recovery was unremarkable and diagnosis of appendicitis was further confirmed on histology.

## Discussion

Acute appendicitis has been recognized as a rare cause of mechanical small bowel obstruction [1,3]. It usually results from adhesion due to periappendicular inflammation and is obviously different from ileus seen in patients with perforated appendicitis presenting with generalized or localized peritonitis. Despite previous reports, this presentation of acute appendicitis is not widely recognized and can result in delays in diagnosis and management.

Harris *et al *[2] (1966) first brought to wider notice the presentation of acute appendicitis as small bowel obstruction and pointed out that very often the differentiation between adynamic ileus and true mechanical obstruction is difficult to elicit clinically. In a series of ten cases analysed by Harris *et al*, all of them had appendicitis with gangrene, necrosis or perforation and the most common cause of the mechanical bowel obstruction was an appendix that lay across the terminal ileum and held down by adhesive bands. In some cases mechanical obstruction was due to the migration of omentum to the right iliac fossa causing kinking of the bowel.

Bose *et al *[4] reported two cases of acute appendicitis presenting with strangulated small bowel. In both cases there was an inflamed appendix wrapping itself around the distal ileum resulting in strangulation of the bowel. Both of these cases were treated with retrograde appendicectomy, one of them requiring a bowel resection as well.

Assenza *et al *[1] reported a case where a patient presented with small bowel obstruction which was found to be due to an inflamed appendix wrapping around the ileum resulting in volvulus and subsequent strangulation. The possible mechanisms for this according to them were adherence of the inflamed tip of the appendix to the posterior peritoneum across the terminal ileum resulting in compression; adherence of the inflamed tip of the appendix to the terminal ileum directly resulting in compression or kinking of a bowel loop; adherence of the inflamed tip of the appendix to the posterior peritoneum forming a loop through which bowel herniates resulting in obstruction and/or strangulation; adherence of the inflamed appendix to the mesentery near the ileocolic artery resulting in subsequent thrombosis and gangrene of the terminal ileum.

However, variations of the above mechanisms have been reported. Zissin *et al *[5] report an unusual case of appendicitis presenting with small bowel obstruction in a patient with intestinal malrotation which was diagnosed pre-operatively by careful analysis of the CT scan, highlighting its use in the diagnosis of bowel obstruction due to appendicitis. Kareem *et al *[6] presented a case of a patient presenting with a six month history of recurrent partial small bowel obstruction with coexisting appendix mass. The diagnosis in this case was only made on surgical exploration and subsequent histopathological examination which revealed findings consistent with an appendicular perforation and non specific ulceration of the surrounding bowel.

Appendicitis can be caused by an appendiceal mucocele and Mourad *et al *[7] report such a case presenting as small bowel obstruction. In their report, a preoperative diagnosis was made by CT scan which revealed a large cystic appendiceal lesion impinging on the caecum. Pitiakoudis *et al *[8] report a case where the patient presented with peritonism and with evidence of small bowel obstruction. Subsequent laparotomy revealed the presence of a ruptured appendiceal mucocele which was successfully treated with an appendicectomy and washout. Histopathological examination in this case indicated a benign ruptured mucocele and pseudomyxoma peritonei.

The presentation of acute appendicitis in the elderly can be atypical which can result in a delayed diagnosis with potential for increased morbidity and mortality [9]. Presentation with mechanical bowel obstruction may pose further challenges. In both our patients, diagnosis was clinically considered and established on CT scan. Despite the numerous mechanisms for acute appendicitis to result in mechanical small bowel obstruction, it is very rarely considered in the differential diagnosis. With the above mentioned reports and our own experience, we would like to highlight the importance of having a high index of suspicion for appendicitis when reviewing patients presenting with small bowel obstruction in the presence of raised inflammatory markers.

## Conclusion

Acute appendicitis should be considered in the differential diagnosis of patients with small bowel obstruction presenting with raised inflammatory markers to avoid delays in management.

## Abbreviations

CT: computerised tomography.

## Consent

Written consent was obtained from both patients for publication of this case report and accompanying images. A copy of the written consent is available for review by the Editor-in-Chief of this journal.

## Competing interests

The authors declare that they have no competing interests.

## Authors' contributions

SH collected the data on both patients and contributed to the literature search and writing of the manuscript. KM conceived the idea and helped with the draft of the case report. DB interpreted the radiological images and contributed to the draft of the manuscript. LB was consultant in charge of patient 1 and contributed to the draft of the manuscript. PKS was the consultant in charge of patient 2 and contributed to the draft of the manuscript. All authors read and approved the final manuscript.
